# Prevalence of visual impairment and eye diseases in Malaysia: A cross-sectional prospective study at the University of Malaya Medical Centre

**DOI:** 10.51866/oa.549

**Published:** 2024-05-15

**Authors:** Xuan Hong Kevin-Tang, Iqbal Tajunisah, Penny Pooi Wah Lott, Sagili Chandrasekhara Reddy

**Affiliations:** 1 MBBS, Masters Ophthal, FRCS, Department of Ophthalmology, University of Malaya, Kuala Lumpur, Malaysia. Email: tajun69@yahoo.com; 2 Final Year Medical Student, Newcastle University Medical Malaysia, Iskandar Puteri, Johor, Malaysia.; 3 MBBS, MS Ophth, FRCOPHTH, Department of Ophthalmology, University of Malaya, Kuala Lumpur, Malaysia.; 4 MBBS, MS Ophth, Department of Ophthalmology, Faculty of Medicine and Defence Health, National Defence University of Malaysia, Kuala Lumpur, Malaysia.

**Keywords:** Cataract, Diabetic retinopathy, Eye diseases, Prevalence, Visual impairment

## Abstract

**Introduction:**

The prevalence of visual impairment and ocular diseases changes over time. This measure can help general practitioners in anticipating common eye disorders that may require ophthalmological referrals to government hospitals. This study aimed to evaluate the prevalence of visual impairment and ocular diseases in an outpatient ophthalmology clinic in a public hospital and the types of investigations frequently conducted to diagnose these diseases.

**Methods:**

A cross-sectional prospective study was conducted over three weeks in the eye clinic of the University of Malaya Medical Centre. The electronic medical records of all patients who attended the outpatient clinic were assessed to collect data on sex, age, type of visit, visual acuity, ocular presentation, investigations conducted and diagnosis of eye diseases. Visual impairment and blindness were categorised as per the World Health Organization criteria.

**Results:**

Among 1002 patients, 327 had visual impairments (32.63%), and nine had blindness (0.9%). Cataracts were the most common ocular disease diagnosed (n=294, 29.74%), followed by glaucoma (n=123, 12.28%) and diabetic retinopathy (n=84, 8.38%). Optical coherence tomography was the most common investigation performed (n=272, 64.9%), followed by Humphrey visual field testing (n=53,12.6%).

**Conclusion:**

Untreated refractive error is the leading cause of visual impairment in children, while cataract, glaucoma and diabetic retinopathy are the main contributors to visual impairment and blindness in elderly individuals. Our study highlights the urgent need for general practitioners to recognise avoidable visual impairment in all age groups to help prevent blindness.

## Introduction

Globally, there are approximately 2.2 billion cases of visual impairment, of which around half are preventable or curable. These cases include presbyopia, refractive errors, cataract and glaucoma.^1^ The number of people with visual impairment is projected to increase by 20% in various age-related ocular conditions such as glaucoma and age-related macular degeneration due to the rapid pace of population ageing.^2^ The projected worsening of visual impairment has notable negative impacts on overall health,^3^ quality of life^4^ and world economy.^5^ Approximately 0.5% of gross domestic product loss and 40 billion dollars of productivity loss are reported in Southeast Asia.^5^

In Malaysia alone, the prevalence of bilateral blindness is estimated to be around 2% across all ages. Seventeen of twenty cases of bilateral blindness are avoidable, and almost 60% are treatable. Cataract remains the commonest cause of blindness (58.6%), followed by diabetic retinopathy (10.4%), other posteriorsegment eye diseases (8.4%) and glaucoma (6.6%).^6^ With the increase in population and life expectancy in Malaysia, the demand for ophthalmological services has grown.^5^ This is proven by the steady two-to-four-fold increment in ophthalmological services in public hospitals in the country.^5,6^

The rising demands, increased awareness of eye diseases and advancement of ophthalmic devices suggest the need for an evaluation of the prevalence of eye diseases and visual impairment in urban populations. Until recently, there is has been a paucity of data on the prevalence of investigations done conducted in ophthalmological centres in Malaysia. Therefore, the investigations ordered for outpatients were recorded for further evaluation and future reference to improve the availability of diagnostic equipment in government hospitals.

The present study aimed to estimate the prevalence of visual impairment and ocular diseases among patients attending the ophthalmic outpatient clinic of the University of Malaya Medical Centre, Kuala Lumpur and determine the number of different investigations conducted to diagnose various eye diseases so that appropriate treatment could be started.

## Methods

This study was as an elective research project of a final-year medical student from Newcastle University Medicine Malaysia conducted from 7 to 25 July 2023 in the ophthalmic outpatient clinic of the University of Malaya Medical Centre. All patients who successfully registered for eye check-up (i.e. new referral, walk-in and follow-up cases) were included. The electronic medical records of all outpatients were accessed, which included their name, registration number, sex, age, visual acuity, type of outpatient visit, ocular presentation, investigations conducted and diagnosis of ocular disease.

Trained nurses assisted with checking and recording the visual acuity of all patients using the Snellen chart placed 6 m from patients, except for extremely young children and patients with significant intellectual disability. Ophthalmologists proceeded with examination using a silt lamp, with qualified optometrists helping with eye refraction.

The recorded visual acuity was categorized according to the World Health Organization (WHO) definition of blindness and visual impairment,^7^ which classifies the findings into five main categories: (i) no visual impairment (visual acuity: 6/6–6/12), (ii) mild visual impairment (visual acuity: <6/12–6/18), (iii) moderate visual impairment (visual acuity: <6/18-6/60), (iv) severe visual impairment (visual acuity: <6/60-3/60) and (v) blindness (visual acuity: <3/60). The vision in the better eye with the best correction was taken for categorising the impairment.^2^ All eye investigations were recorded even when there was more than one eye investigation performed for patients.

Some patients had more than one existing eye disease. Each patient’s ocular presentation was recorded according to the subspecialty clinic they presented to, as per the International Statistical Classification of Diseases.

The Statistical Package for the Social Sciences version 21.0 (IBM SPSS Statistics, Chicago, Illinois, USA) was used to analyse all data obtained. The means and standard deviations of age and total numbers of various categories of visual impairment and eye diseases were calculated. The frequency of all variables was expressed as percentages.

## Results

During the study period, the electronic medical records of a total of 1002 patients were reviewed. Of the patients, more than half were women (n=544, 54.3%), and the rest were men (n=458, 45.7%). The mean age of the study population was 73.2±2.29 years (range=1 month to 93 years). Most outpatient visits were for follow-up cases (n=703, 70.2%) and the remaining for new cases (n=299, 29.9%). Of the new cases, 164 (54.58%) were walk-in cases, while 135 (45.15%) were referred from either optometrists, primary care clinics or the trauma and emergency unit.

More than half of the patients (60.98%) were noted to have no visual impairment, while 0.9% were diagnosed with blindness. The visual acuity of 37 patients could not be assessed either because they were extremely young or because they had significant cognitive and physical disabilities that prevented accurate visual assessments at the time of their visit.

The patients aged 61-80 years constituted the largest population attending the eye clinic (n=585, 58.38%). Those aged 61-70 and 7180 years comprised the largest groups that had moderate-to-severe visual impairment (6.19% and 6.09%, respectively).

The study population presented different grades of visual impairment (as per the WHO classification of visual acuity) according to their sex and age (Table 1).

**Table 1:** Different grades of visual impairment in both sexes and different ages (N=1002).

Variable	Cases, n (%)	Visual acuity					
		No visual impairment (6/6-6/12), n (%)	Mild visual impairment (<6/12-6/18), n (%)	Moderate visual impairment (<6/18-6/60), n (%)	Severe visual impairment (<6/60-3/60), n (%)	Blindness (<3/60), n (%)	Not available, n (%)
**Total**	1002 (100)	611 (60.98)	123 (12.30)	188 (18.80)	16 (1.60)	9 (0.90)	55 (5.5)
**Sex**							
Male	458 (45.71)	277 (27.64)	67 (6.69)	73 (7.29)	6 (0.60)	5 (0.50)	30 (2.99)
Female	544 (54.29)	334 (33.33)	56 (5.59)	115 (11.48)	10 (1.00)	4 (0.40)	25 (2.50)
**Age**							
0–10 years	60 (5.99)	18 (1.80)	1 (0.10)	4 (0.40)	0 (0.00)	0 (0.00)	37 (3.69)
11–20 years	24 (2.40)	19 (1.90)	2 (0.20)	3 (0.30)	0 (0.00)	0 (0.00)	0 (0.00)
21–30 years	37 (3.69)	27 (2.69)	4 (0.40)	3 (0.30)	1 (0.10)	0 (0.00)	2 (0.20)
31–40 years	47 (4.69)	37 (3.69)	3 (0.30)	5 (0.50)	1 (0.10)	2 (0.20)	1 (0.10)
41–50 years	54 (5.39)	40 (3.99)	4 (0.40)	7 (0.70)	0 (0.00)	1 (0.10)	1 (0.10)
51–60 years	118 (11.78)	75 (7.49)	14 (1.40)	24 (2.40)	1 (0.10)	2 (0.20)	3 (0.30)
61–70 years	308 (30.74)	185 (18.46)	47 (4.69)	62 (6.19)	6 (0.60)	4 (0.40)	6 (0.60)
71–80 years	277 (27.64)	168 (16.77)	36 (3.59)	61 (6.09)	4 (0.40)	0 (0.00)	4 (0.40)
81–90 years	70 (6.99)	37 (3.69)	11 (1.10)	18 (1.80)	3 (0.30)	0 (0.00)	1 (0.10)
90–100 years	7 (0.70)	5 (0.10)	1 (0.10)	1 (0.10)	1 (0.10)	4 (0.40)	0 (0.00)

More than half of the study population (n=541, 53.99%) did not report any active/serious symptoms during the outpatient visits, as they were mostly follow-up patients. Blurring of vision was the most common ocular presentation in 219 (21.86%) patients. There were various ocular presentations noted such as red eyes in 35 (3.49%) patients, black spots in front of the eyes (floaters) in 30 (2.99%) patients, lid swelling in 25 (2.49%) patients and dry eyes in 22 (2.19%) patients. Foreign body sensation, headache, glaring, sticky eyes, gritty eyes, squinting and diplopia were the other common complaints on presentation.

The visual acuity was not recorded in extremely young children or children with advanced physical handicap, while the reading was rarely missing in some adult patients.

The prevalence of ocular diseases among the study population is shown in Table 2. Diseases of the lens and retina and glaucoma were most commonly seen. This was because a significant proportion of the study population had cataract (n=213, 21.26%), other diseases of the lens and glaucoma (n=123, 12.28%) and diabetic retinopathy including maculopathy (n=84, 8.38%) and other retinal diseases. Cataract was the primary contributor to severe visual impairment in 4 out of 16 patients (25.0%), whereas glaucoma was the primary cause of blindness in three out of nine patients (33.33%). Conversely, 79 out of 219 patients (36.07%) who reported blurring of vision and 112 out of 541 patients (20.70%) who reported no disturbing eye complaints were found to have cataract.

**Table 2:** Prevalence of ocular diseases among the study population (N=1002).

Ocular disease	n (%)		n (%)
**Conjunctiva**	**47 (4.69)**	**Vitreous**	**32 (3.19)**
Conjunctivitis	34 (3.39)	Endophthalmitis	1 (0.10)
Corneal abrasion	1 (0.10)	Posterior vitreous detachment	21 (2.10)
Corneal ulcer	1 (0.10)	Vitreous haemorrhage	10 (1.00)
Pterygium	7 (0.70)	**Uveal tract**	27 (2.69)
Subconjunctival haemorrhage	4 (0.40)	Vogt–Koyanagi–Harada disease	1 (0.10)
**Cornea**	**38 (3.79)**	Central serous chorioretinopathy	2 (0.20)
Band keratopathy	2 (0.20)	Ocular sporotrichosis	1 (0.10)
Corneal ulcer	1 (0.10)	Ocular toxoplasmosis	1 (0.10)
Corneal abrasion	7 (0.70)	Toxic anterior syndrome	1 (0.10)
Corneal decompensation	14 (1.40)	Uveitis	20 (2.00)
Corneal ulcer	1 (0.10)	**Retina**	**207 (20.66)**
Herpes zoster ophthalmicus	2 (0.20)	ARMD	32 (30.19)
Keratitis	10 (1.00)	Vitreomacular traction syndrome	1 (0.10)
Keratoconus	1 (0.10)	CRAO	1 (0.10)
**Eyelids**	**38 (3.79)**	CRVO	12 (1.20)
Blepharitis	9 (0.90)	Cystoid macular oedema	6 (0.60)
Chalazion	12 (1.20)	Diabetic maculopathy	31 (3.09)
Dermoid cyst	1 (0.10)	Diabetic retinopathy	53 (5.29)
Ectropion	1 (0.10)	Epiretinal membrane	29 (2.89)
Epiblepharon	1 (0.10)	Hypertensive retinopathy	1 (0.10)
Hordeolum	1 (0.10)	IPCV	3 (0.30)
Involutional ptosis	1 (0.10)	Lamellar hole	2 (0.20)
Lagophthalmos	1 (0.10)	Lattice degeneration	1 (0.10)
Lid meibomitis	8 (0.80)	Macular hole	5 (0.50)
Lid oedema secondary	1 (0.10)	Retina	207 (20.66)
to food allergy	1 (0.10)	Macular oedema	3 (0.30)
Trichiasis	2 (0.20)	Peripapillary atrophy	1 (0.10)
Glaucoma	124 (12.38)	Retinal detachment	10 (1.00)
**Glaucoma**	**123 (12.28)**	Retinal haemorrhage	1 (0.10)
Pigment dispersion syndrome	1 (0.10)	Retinal hole	6 (0.60)
**Lacrimal system**	**11 (1.10)**	Retinal tear	2 (0.20)
Dacryocystitis	4 (0.40)	Retinitis pigmentosa	2 (0.20)
Dry eyes	3 (0.30)	Retinopathy of prematurity	2 (0.20)
Nasolacrimal duct obstruction	3 (0.30)	Retinoschisis	1 (0.10)
Sicca syndrome	1 (0.10)	Subretinal neovascularisation	2 (0.20)
**Lens**	**298 (29.74)**	**Eye trauma**	**7 (0.70)**
Cataract	213 (21.26)	Corneal foreign body	2 (0.20)
Lens dislocation	3 (0.30)	Eye evisceration	1 (0.10)
Lens subluxation	3 (0.30)	Hyphaemia	1 (0.10)
Pseudophakia	76 (7.58)	Ocular trauma	1 (0.10)
Posterior capsular opacification	3 (0.30)	Orbital trauma	2 (0.20)
**Optic nerve and visual pathway**	**14 (1.40)**	**Orbit**	**28 (2.79)**
Alternating esotropia	1 (0.10)	Myasthenia gravis	2 (0.20)
Colour blindness	1 (0.10)	Orbital wall fracture	2 (0.20)
Cranial nerve palsy	4 (0.40)	Periorbital haematoma	3 (0.30)
Optic neuropathy	2 (0.20)	Periorbital cellulitis	1 (0.10)
Pituitary tumour	1 (0.10)	Pre-septal cellulitis	7 (0.70)
Space-occupying lesion	1 (0.10)	Socket contracture	1 (0.10)
Toxoplasma oculopathy	2 (0.20)	Thyroid eye disease	12 (1.20)
Posterior communicating artery aneurysm	1 (0.10)	**Normal**	**103 (10.28)**
Optic neuropathy	1 (0.10)	**Refractive error**	**16 (1.60)**
		Myopia	10 (1.00)
		Hyperopia	5 (0.50)
		Astigmatism	1 (0.10)

CRAO, central retinal artery occlusion; CRVO, central retinal vein occlusion; IPCV, idiopathic polypoidal choroidal vasculopathy; ARMD, age related macular degeneration.

More than half of the patients (n=551, 53.29%) underwent only the visual acuity test and slit lamp examination in the eye clinic, without additional investigations to diagnose their ocular conditions. In addition to the two tests, various types of ophthalmological investigations (n=4l9) were conducted in the eye clinic to support the diagnosis of the ocular diseases (Table 3). Optical coherence tomography was the most common investigation ordered (n=272, 64.9%), followed by Humphrey visual field (n=53, 12.6%) and refraction testing (n=50, 11.9%).

**Table 3:** Investigations conducted for the diagnosis of various eye diseases (n=4l9).

Eye investigation	n (%)
Optical coherence tomography	272 (64.9)
Humphrey visual field testing	53 (12.6)
Refraction testing	50 (11.9)
Hess chart	17 (4.0)
Fundus photography	12 (2.8)
Fundus fluorescein angiography	7 (1.6)
B-scan ultrasonography	5 (1.2)
Pentacam corneal topography	2 (0.4)
Specular microscopy	1 (0.2)

A total of 419 investigations were performed for a more accurate diagnosis of the eye diseases during the 3-week study period (Table 3).

## Discussion

The University of Malaya Medical Centre provides eye care to the population of the federal territory of Kuala Lumpur and patients referred from surrounding eye care centres. There are other government and private tertiary eye care centres that also provide eye care for these populations. The estimated population of Kuala Lumpur in July 2023 was 2 million.^8^ Given the rapid rate of urbanisation, this population is set to grow by about 2% per year. This means that the burden of age-related ocular diseases,^9^ such as cataract, glaucoma and diabetic retinopathy, is expected to rise in the urban population.

In this study, we found that 20.4% of the study population had moderate-to-severe visual impairment (<6/18–6/60) in their best corrected vision. A comparison of the percentage of visual impairment and blindness between the present study and other studies conducted in Malaysia is shown in Table 4.

**Table 4:** Comparison of the percentage of visual impairment and blindness between the present study and other studies conducted in Malaysia.

Location in Malaysia	Sample size	Age	Moderate-to-severe visual impairment	Blindness
Kuala Lumpur (urban)^10^	1169	1 month to 100 years	9.8%	0.9%
Sepang district (rural)^11^	341	40 years and above	18.9%	0.7%
Kuala Lumpur (urban)^12^	1322	55 years and above	9.0%	2.1%
Temerloh (rural)^13^	1081	1 month to 100 years	9.3%	3.20%
Entire Malaysia (NES II)^14^	2284	50 years and above	6.4%	1.2%
Selangor^15^ (urban and rural)	230	60 years and above	27.3%	Not available
PRESENT STUDY (urban)	1002	1 month to 93 years	20.4%	0.9%

A similar study was conducted in the same hospital 15 years back in 2008^10^ to determine the prevalence of visual impairment and ocular diseases. The incidence of visual impairment has now increased to 20.4% from 9.8% observed in 2008. The prevalence of visual impairment is higher in the present study than in most other studies, except for the study in Selangor, which included both urban and rural areas. This might be because our study site is one of the largest specialised tertiary referral centres that receive more complex referral cases with high levels of visual impairment from all around the nation.

In our study, the prevalence of moderate-to-severe visual impairment was much higher among the women (12.48%) than among the men (7.89%). The prevalence of moderate-to-severe visual impairment was proportional with advancing age. The patients aged 60 years and above accounted for 15.88% of the moderate-to-severe visual impairment cases (Table 1). This was in correspondence to the large number of cataracts, age-related macular degeneration, glaucoma and diabetic retinopathy cases. The ageing process impairs the clearance of cellular by-products, and the production of reactive oxygen species and reduction of antioxidants in ocular tissues lead to age-related ocular diseases such as cataract, glaucoma and macular degeneration.^16,17^ The relatively smaller number of cases of refractive errors in our study (1.6%) than in a previous study (10.8%)^10^ might be attributed to the increased awareness of refractive errors within the urban population due to their higher level of education, the availability of nearby optometrists who could prescribe glasses and the availability of more eye care services over time in the private sector.

In this study, 0.9% of the patients had blindness in their best corrected vision. This finding is similar to the report by Reddy et al.^10^ and the National Eye Survey II.^14^ In contrast, the prevalence of blindness is lower than that reported in another study conducted in Kuala Lumpur,^12^ which is most probably due to the difference in the categorisation of visual acuity-based blindness. The present study defined blindness in accordance with the WHO definition, which is less than 3/60 vision in the better eye with the best corrected vision. Conversely, Jamaluddin Ahmad et al.12 interpreted any visual acuity less than 6/60 as blindness. Our study also noted a lower prevalence of blindness in comparison to that in a district hospital in Temerloh in Pahang state.^13^ This is likely because of the increased awareness of avoidable blindness in the urban population, better access to ophthalmological services in urban areas and higher socioeconomic status, which enables affording glasses and surgical interventions.^18^

More than half of our study population (53.99%) did not report any active/serious eye complaint. This could be explained by the large number of follow-up cases in the clinic, of which most were mild cataract that required yearly monitoring. Our study also found that 10.3% of the patients had normal vision, being referred from other medical departments for annual eye screening for drug toxicity and underlying systemic diseases.^19-21^

Optical coherence tomography remained the most popular investigation ordered. This could support the diagnosis of glaucoma and retinal diseases. In general, optical coherence tomography helps to monitor changes in the nerve fibres at the retina to allow for timely intervention, which is in line with the good clinical practice guideline.^22,23^

### Limitations

Due to the small sample size, short data collection period in a single tertiary eye care centre and geographical limitation, the enrolled patients might not fully reflect the full range of visual impairments and ocular conditions in the urban population of Malaysia.

## Conclusion

In summary, patients aged 60-80 years comprise the largest age group attending ophthalmic outpatient clinics. Cataract, glaucoma, age-related macular degeneration and diabetic retinopathy account for most cases of visual impairment and blindness within the elderly population, while uncorrected refractive errors are the commonest ocular condition and visual impairment in children. The increasing prevalence of avoidable visual impairment is a wake-up call for more specialised training, workforce expansion and development of eye care centres that offer affordable treatment in urban areas in Malaysia. Further, more resources are needed to purchase related equipment to enhance the treatment and care for patients with posterior-segment eye diseases.

## Data sharing statement

Data are available upon request.

**How does this paper make a difference in general practice?**

- A concise overview of the prevalence of visual impairment and eye diseases commonly seen in eye clinic attending an eye clinic in a public hospital in an urban town in Malaysia is provided
- The study also reports the most common investigations ordered in a busy clinic to achieve a diagnosis.
- The findings could help general practitioners in determining what type of cases should be referred to eye specialist and the investigations patients need to achieve their diagnosis.
